# Characteristics and prognosis of patients with cryptogenic stroke and suggestive of patent foramen ovale

**DOI:** 10.1186/s12947-021-00255-0

**Published:** 2021-06-05

**Authors:** Jaehuk Choi, Min-Kyung Kang, Jin-Sun Jun, Dong Geum Shin, Donghoon Han, Seonghoon Choi, Jung Rae Cho, Namho Lee

**Affiliations:** 1 Division of Cardiology, Dongtan Sacred Heart Hospital, Hallym University Medical Center, Seoul, South Korea; 2 Division of Cardiology, Kangnam Sacred Heart Hospital, Hallym University Medical Center, Seoul, South Korea; 3 Division of Neurology, Kangnam Sacred Heart Hospital, Hallym University Medical Center, Seoul, South Korea

**Keywords:** Cryptogenic stroke, Patent foramen ovale, Agitated saline echocardiography

## Abstract

**Aims:**

The purpose of this study were to identify the usefulness of screening for PFO using agitated saline echocardiography (ASE) and characteristics and prognosis of patients with suggestive of patent foramen ovale (PFO).

**Methods:**

Three hundred three patients (mean age, 53 ± 9 years; 199 [66%] men) admitted with acute stroke or suspicion of stroke were included. Patients were classified into those with and without right-to-left shunt (RLS) according to the ASE results (positive ASE [*n* = 92] vs. negative ASE [*n* = 211]). Fifty-one out of ninety-two patients with positive ASE and twenty-one out of two hundred eleven patients with negative ASE underwent TEE with ASE to confirm PFO.

**Results:**

Ninety-two were positive for ASE and thirty-six of the fifty-one patients who underwent TEE were confirmed as having PFO. Of the patients with RLS grade 1, 50% were diagnosed with PFO and all patients with RLS grade ≥ 2 were diagnosed with PFO. All patients with negative ASE had no PFO (sensitivity of 100% and specificity of 58%). Patients with positive ASE were younger, had a lower body mass, and a lower prevalence of hypertension. The positive ASE patients had a higher mean Sʹ velocity and better diastolic function. Four of ninety-one patients with positive ASE and thirteen of one hundred seventy-seven showed recurrence of stroke and suspicion of stroke.

**Conclusion:**

Transthoracic ASE is a good method to screen for PFO. Patients with suggestive of PFO had lower risk factors, less atherosclerosis, and better cardiac performance.

## Introduction

Patent foramen ovale (PFO) has been known to have significant association with cryptogenic ischemic stroke [1]. Despite optimal medical therapy, the rate of stroke recurrence in PFO is estimated to be 25% within a 4-year period [2]. Since the development of percutaneous device closure of PFO as a potential therapeutic option in this group of patients, PFO closure is known to be beneficial as compared to medical therapy in the preventions of recurrent neurological events [3]. The role of echocardiography is very important both in the diagnosis of PFO and in the evaluation of PFO for transcatheter closure [4, 5]. Transesophageal echocardiography (TEE) is considered the gold standard for the diagnosis of PFO because it provides anatomical details [6]. However, TEE requires an expert echocardiographer and is poorly tolerated by patients (more than 40% patients refused TEE in our hospital). In addition, Maggiore, P et al. published an article concerns about TEE with bubble study and the need to diagnose PFO in patients with ischemic stroke [7]. Transcranial Doppler (TCD) might be an alternative method to overcome these limitations of TEE [8]. However, TCD also is an examiner-dependent method, and cannot be applied in all patients due to difficulties with ultrasound penetration through the skull [9]. Contrast echocardiography using agitated saline contrast is good for detection of right-to-left shunts (RLS) [10]. TEE with agitated saline contrast is a gold standard for diagnosis of PFO for its direct visualization of PFO slit and confirmation of paradoxical RLS. However, sometime it is impossible for TEE to be performed in all patients whom PFO should be screened due to abovementioned pitfalls of TEE. However, transthoracic echocardiography (TTE) with agitated saline contrast (agitated saline echocardiography; ASE) can be used to detect RLS in patients with suspected cryptogenic stroke without any need for expert sonographer or patient’s sedation. Therefore, this study aimed to evaluate the usefulness of transthoracic ASE for screening of PFO and clinical characteristics of suggestive PFO in patients with cryptogenic stroke in South Korea.

## Methods

This study was a cross-sectional, observational study. In this study, three hundred-three patients [mean age: 53 ± 9, 199 (66%) men) who attended the Kangnam Sacred heart Hospital, Hallym University from the April, 2016 to May, 2018. All patients were referred to division of cardiology for evaluation of cardiac embolic source. Patients with age < 65 and with acute stroke, transient ischemic attack (TIA), or retinal vessel occlusion were included. All patients had undergone TTE, carotid ultrasound, ankle-brachial index (ABI), pulse wave velocity (PWV), 24-h-Holter monitoring, and ASE. We also collected participant data on demographic, anthropometric, and laboratory parameters including cardiac biomarkers. Patients with significant valvular diseases, myocardial infarction, atrial fibrillation (AF), intra-cardiac mass (such as thrombi, myoxoma, papillary fibroelastoma, vegetation, et al.), presence of intra-cardiac shunt except PFO, peripheral arterial disease (PAD), significant obstruction (diameter stenosis ≥ 75%) of carotid arteries, or brain hemorrhage were excluded from this study. Study population were sorted into two groups – positive ASE (*n* = 92) vs. negative ASE (*n* = 211). In addition, TEE was performed to 72 consenting patients to confirm PFO (51 patients with positive ASE and 21 patients with negative ASE agreed to be performed TEE). We observed whether there was a recurrence of stroke or suspicion of stroke in both groups for up to 2 years.

### Echocardiography

TTE was performed using standard techniques with a 2.5-MHz transducer. The standard 2-D and Doppler echocardiography was performed using a commercially available echocardiographic machine (Vivid 7R GE Medical System, Horten, Norway) with the same setup interfaced with a 2.5-MHz phased-array probe. All measurements were performed according to the guideline [11]. With the study participant in the partial left decubitus position and breathing normally, the observer obtained images from the parasternal long and short axes and from the apical four chamber and two-chamber and long-axis views. Depth setting was optimized to display the LV on the screen as large as possible and the same field depth was kept for both four and two-chamber apical views. Sector width was reduced to increase spatial and temporal resolution. Left ventricular end-diastolic dimensions (LV EDD), end-diastolic interventricular septal thickness (IVSTd), and end-diastolic LV posterior wall thickness (PWTd) were measured at end-diastole according to the standards established by the American Society of Echocardiography. LV ejection fraction (EF) was determined by the biplane Simpson’s method. Maximal left atrial (LA) volume was calculated using the Simpson method and indexed to the body surface area. LV mass was calculated using the Devereux formula = 1.04[(LVEDD + IVSTd + PWTd)^3^ − (LVEDD)^3^] − 13.6. Thereafter, the LV mass index (LVMI) was calculated and indexed to body surface area.

Mitral flow velocities were recorded in the apical four-chamber view. Mitral inflow measurements included the peak early (E) and peak late (A) flow velocities and the E/A ratio. The tissue Doppler of the mitral annulus movement was also obtained from the apical four-chamber view. A 1.5-mm sample volume was placed sequentially at the septal annular sites. The analysis was performed for early diastolic (E’), late diastolic (A’), and systolic (S’) peak tissue velocities. As a noninvasive parameter for LV stiffness, the LV filling index (E/E’) was calculated by the ratio of transmitral flow velocity to annular velocity. Adequate mitral and tissue Doppler image (TDI) signals were recorded in all patients.

The mean longitudinal global strain (GS) of LV was calculated from the apical 4,3,2-chamber views by speckle-tracking 2D-strain imaging [12].

### Agitated Saline Echo (ASE)

**Fig. 1:**
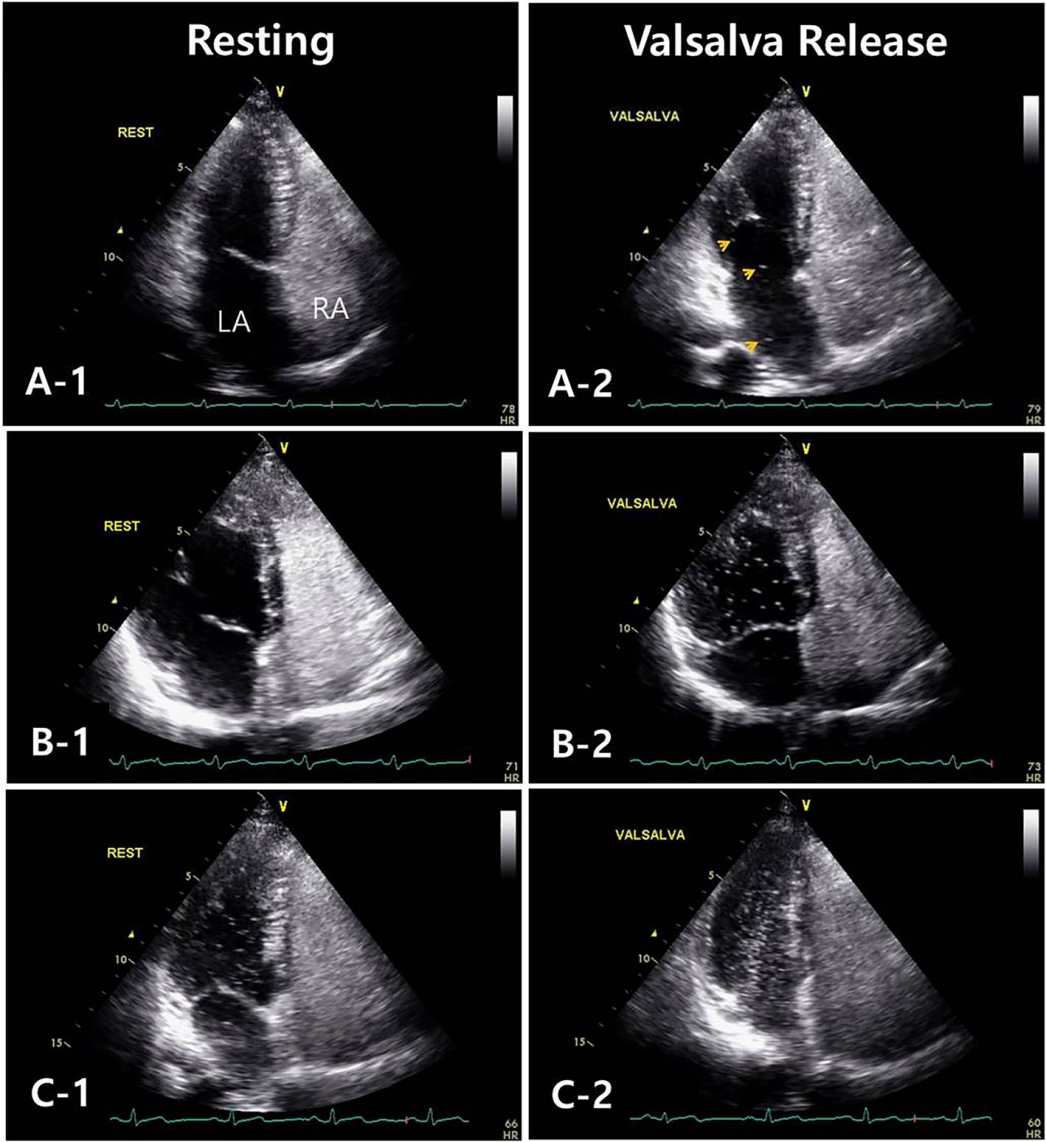
**A-1,2:** Grade 1—< 5 bubbles within LV only after Valsalva maneuver (yellow arrows: bubbles within LA and LV) LA: left atrium, LV: left ventricle. **B-1,2:** Grade 2—5 to 25 bubbles within LV both resting and after Valsalva maneuver. **C-1,2:** Grade 3—> 25 bubbles or opacification within LV

Nine millilitre of normal saline agitated with one millilitre of room air, agitated back and forth between two sterile syringes just before intra-venous bolus injection through a forearm vein to detect right to left shunt (RLS) [13]. The injections performed at rest and provocative maneuver (Valsalva maneuver in this study) to increase the right atrial (RA) pressure. The presence of PFO is presumed 3 to 5 cardiac cycles after complete opacification of the RA. We modified the protocol used for shunt grading incorporated 4 grades [8]: grade 1: < 5 bubbles; grade 2: 5 to 25 bubbles; grade 3: > 25 bubbles; grade 4: opacification of chamber to 3 grades: grade 1: < 5 bubbles only after Valsalva maneuver; grade 2: 5 to 25 bubbles both resting and after Valsalva maneuver; grade 3: > 25 bubbles or opacification (Fig. 1).

### Carotid ultrasound

A high-resolution B-mode ultrasound (Vivid 7R GE Medical Systems, Horten, Norway) equipped with a 7.5-MHz linear array transducer was used for carotid ultrasonography. In the longitudinal view, carotid intima-media thickness (IMT) was determined as the distance from the media adventitia interface to the intima lumen interface on the far wall in a region free of plaque [14]. The examiner assessed the presence of carotid plaques, which were defined as focal structures that encroached into the lumen by at least 100% of the surrounding IMT value. Common carotid artery IMT (CCA-IMT) was measured between the origin of the carotid bulb and a point 10 mm proximal to the CCA, and the carotid bulb IMT (CB-IMT) was measured in the carotid bulb region. CCA-IMT and CB-IMT values were determined as the average of the maximum IMT of the left and right CCA and CB.

### Pulse Wave Velocity (PWV)

We measured PWV to estimate arterial stiffness, which is generally accepted as the most simple, non-invasive, and validated indicator of arterial stiffness [15]. PWV was measured using a VP-2000 automated device (Colin Co., Komaki, Japan). The right and left brachial-ankle PWV (baPWV) were simultaneously measured. The patients were placed in a supine position about 15 min prior to the test. The pressure waveforms of the brachial and tibial arteries were obtained from the occlusion and monitoring cuffs wrapped around the upper arm and lower leg. All measurements were performed in a quiet, temperature-controlled room (22 ± 1 °C), with the patients having fasted overnight. The baseline brachial systolic and diastolic blood pressure (BP), heart rate (HR), and PWV were simultaneously measured.

### Statistical analysis

All continuous data are expressed as mean ± SD, and all categorical data are presented as percentage or absolute numbers. Continuous variables were analyzed using Student’s t-test and dichotomous variables were analyzed using the chi square test. Data was statistically analyzed using SPSS version 20, and *p*-value < 0.05 was considered statistically significant for all the analyses. Significant factors were tested in a univariate binary logistic regression analysis, and then only significant variables were entered in a stepwise multivariate logistic regression analysis to identify the independent predictors for the positive ASE. In addition, multivariate analysis (logistic regression) was performed.

### Ethics approval and consent to participate

All procedures performed in studies involving human participants were in accordance with the ethical standards of the institutional and/or national research committee and with the 1964 Helsinki declaration and its later amendments or comparable ethical standards. The local institutional review board approved this study (IRB file No.: HKS 2018–01-014). Informed consent was not obtained from all individual participants included in the study, because this study was a cross-sectional, observational study.

## Results

### Clinical parameters of the study population

Of the 303 patients, 92 were positive ASE, while 36 of 51 (71%) patients who underwent TEE were confirmed as having PFO. Among the patients with grade 1 RLS, 50% were diagnosed with PFO; in contrast, all patients with RLS grade ≥ 2 RLS were diagnosed with PFO (Table 1). On the other hand, no patients with negative ASE were diagnosed with PFO on TEE (sensitivity of 100% and specificity of 58%). The patients’ clinical characteristics are shown in Table 2. The study population included 303 patients (mean age, 53 ± 9 years; 199 [66%] men) who were admitted for acute stroke or suspicion. Patients with positive ASE were significantly younger and had lower body mass index (BMI). The prevalence of hypertension was also lower in patients in the positive ASE group. Brain magnetic resonance imaging (MRI) showed a similar frequency of acute stroke or TIA. However, the incidence of any abnormality on brain MRI including undetermined lesions was lower in patients with positive ASE; also, patients with positive ASE were much less likely to use aspirin. Risk of paradoxical embolism (RoPE) score [16] was higher in patients with positive ASE. 91 of 92 (99%) patients with positive ASE and 177/211 (84%) patients with negative ASE were followed for up to 2 years. Among them, 4 of 91 (4%) patients with positive ASE and 13 of 177 (7%) showed recurrence of stroke and suspicion of stroke. All 16 patients except 1 of the relapsed patients were taking antiplatelet drugs (3 were aspirin; 4 were clopidogrel, 7 were aspirin and clopidogrel, 1 was cilostazol, 1 was trifusal). Device therapy was performed in 6 patients with PFO confirmed by TEE, and all were ASE grade 3. Medical treatment, including antiplatelet drugs, was performed in all individual patients.

**Table 1:** The results of transesophageal echocardiography

PFO	ASE ( +), 51		ASE (-), 21		*p*
Yes	36 (71%)		0 (0%)		< 0.001
no	15 (29%)		21 (100%)		
	Shunt grade				
	Gr 0 (21)	Gr 1, (30)	Gr 2 (7)	Gr 3 (14)	*p*
Yes	0 (0%)	15 (50%)	7 (100%)	14 (100%)	< 0.001
no	21 (0%)	15 (50%)	0 (0%)	0 (0%)	

*PFO* patent foramen ovale, *ASE* agitated saline echo (transthoracic)

**Table 2:** Clinical parameters of the study population

	Positive ASE (*n* = 92)	Negative ASE (*n* = 211)	*p*
Age (years)	49.5 ± 10.8	53.8 ± 8.1	0.001
Male gender	60 (65.0%)	139 (65.9%)	1.000
SBP (mmHg)	124.9 ± 17.2	127.3 ± 16.2	0.257
DBP	76.8 ± 12.5	76.9 ± 10.4	0.971
Heart rate (bpm)	69.3 ± 11.2	67.2 ± 10.4	0.108
Body mass index (kg/m^2^)	24.1 ± 3.3	25.0 ± 3.9	0.046
Brain MRI			0.331
TIA	18 (19.6%)	25 (11.8%)	
Acute stroke	53 (57.6%)	146 (69.2%)	
Small vessel disease	8 (8.7%)	17 (8.1%)	
Undetermined	13 (14.1%)	20 (10.9%)	
Any abnormality	61 (66.3%)	163 (77.3%)	0.046
Brain MRA			0.282
Normal	41 (48.8%)	85 (40.5%)	
Aneurysm	14 (16.7%)	363 (17.1%)	
Stenosis	31 (36.9%)	101 (48.1%)	0.092
Vasculitis et al	5 (6.0%)	7 (3.3%)	
Thrombolysis	2 (1.7%)	10 (5.6%)	0.133
Hypertension	28 (30.4%)	92 (43.6%)	0.041
Diabetes	15 (16.3%)	46 (21.8%)	0.350
Prior events	8 (8.7%)	26 (12.3%)	0.432
RoPE score	5.73 ± 1.51	4.99 ± 1.54	< 0.001
Current smoker	43 (46.7%)	95 (45.0%)	0.803
Medications			
Aspirin	1 (1.1%)	18 (8.6%)	0.010
RASB	15 (16.3%)	40 (19.0%)	0.629
Statin	8 (8.7%)	28 (13.3%)	0.335
^a^PFO device therapy	6 (6.6%)	0	0.001
Recurrence	4 (4.7%)	13 (6.2%)	0.407
Loss of follow up	1(1.1%)	34 (16.1%)	0.001

Data are mean ± standard deviation (SD) or n (%)

*ASE* agitated saline echo, *SBP* systolic blood pressure, *DBP* diastolic BP, *MRI* magnetic resonance image, *TIA* transient ischemic attack, *MRA* magnetic resonance angiography, *RASB* Renin-Angiotensin system blocker, *CCB* calcium-channel blocker, *OHA* oral hypoglycemic agents, *PFO* patent foramen ovale

^a^Device therapy was performed in 6 patients with PFO confirmed by transesophageal echocardiography, and all were ASE grade 3

### Echocardiographic parameters of the study population

Table 3 shows the patients’ echocardiographic parameters; no significant intergroup differences were seen in cardiac size or LV systolic function. However, patients with positive ASE had higher Sʹ velocity and better diastolic function (higher Eʹ and Aʹ velocities and more favorable LV filling pattern). In addition, LV dimension and LVMI were slightly smaller and LV EF was slightly higher in patients with positive ASE with marginal statistical significance.

**Table 3:** Echocardiographic parameters of the study population

	Positive ASE (*n* = 92)	Negative ASE (*n* = 211)	*p*
LAVI (ml/m^2^)	23.5 ± 7.0	23.9 ± 7.3	0.695
LV SWTd (mm)	9.6 ± 1.8	9.9 ± 1.7	0.199
LV PWTd	9.4 ± 1.7	9.7 ± 1.6	0.177
LV EDD (mm)	48.5 ± 4.2	49.4 ± 4.0	0.071
LV ESD	30.9 ± 3.4	31.7 ± 4.0	0.069
LVMI (g/m^2^)	96.1 ± 26.5	101.5 ± 23.8	0.078
LV EF (%)	65.5 ± 4.9	64.5 ± 4.9	0.108
GS (%)	-17.8 ± 3.2	-18.0 ± 3.0	0.636
E (cm/s)	68.3 ± 18.3	68.4 ± 16.7	0.945
A (cm/s)	67.9 ± 18.9	71.2 ± 17.1	0.134
E/A ratio	1.10 ± 0.41	1.02 ± 0.39	0.105
DT (ms)	199.6 ± 39.2	208.0 ± 43.5	0.116
E’ (cm/s)	8.2 ± 3.0	7.5 ± 2.8	0.031
A’ (cm/s)	9.6 ± 2.1	9.1 ± 1.7	0.020
E’/A’	0.9 ± 0.4	0.9 ± 0.4	0.607
E/E’	9.3 ± 4.3	10.0 ± 3.3	0.123
S’ (cm/s)	8.0 ± 1.7	7.3 ± 1.4	< 0.001
RVSP	27.4 ± 6.1	27.4 ± 5.4	0.378
Diastolic grade			0.012
normal	40 (43.5%)	71 (34.3%)	
Grade 1	47 (51.1%)	134 (64.7%)	
Grade 2	5 (5.6%)	2 (1.0%)	

Data are represented as mean ± SD or n (%)

*LAVI* left atrial volume index, *LVMI* left ventricular mass index, *SWTd* diastolic septal wall thickness, *SWTs* systolic SWT, *PWTd* diastolic posterior wall thickness, *sPWT* systolic PWT, *DWS* diastolic wall strain, *LV EDD and ESD* LV end-diastolic and systolic dimension, *EF* ejection fraction, *GS* global strain, *DT* deceleration time, *RVSP* right ventricular systolic pressure

### Laboratory findings and other parameters

Table 4 shows laboratory parameters and carotid ultrasound results. There were no significant intergroup differences in blood test results. The values of PWV, an indicator of arterial stiffness, were lower in patients with positive ASE. In addition, IMT was lower and the presence of carotid plaque was significantly lower in patients with positive ASE. However, there was no significant intergroup difference in maximal thickness of the carotid plaques.

**Table 4:** Laboratory and other parameters

	Positive ASE (*n* = 92)	Negative ASE (*n* = 211)	*p*
D-dimer (μg/dL)	0.18 ± 0.05	0.28 ± 0.28	0.487
Serum creatinine (mg/dL)	0.90 ± 0.82	0.86 ± 0.67	0.695
GFR (mL/min/1.73m^2^)	84.1 ± 14.1	83.8 ± 12.8	0.869
Total cholesterol (mg/dL)	185.4 ± 38.2	193.2 ± 40.0	0.119
LDL (mg/dL)	112.8 ± 37.6	117.4 ± 33.1	0.314
HDL	46.7 ± 14.2	45.8 ± 13.5	0.607
BNP (pg/mL)	51.9 ± 86.9	37.6 ± 45.8	0.191
Troponin I (ng/mL)	0.011 ± 0.026	0.007 ± 0.09	0.359
^a^TG (mg/dL)	115 (30–458)	135 (24–1575)	0.401
^a^CK-MB (ng/mL)	1.05 (0.18–20.39)	1.15 (0.02–34.19)	0.782
PWV (m/s)	1.62 ± 0.31	1.72 ± 0.37	0.034
IMT (mm)	0.66 ± 0.19	0.74 ± 0.23	0.007
Carotid plaque	51 (61%)	160 (76%)	0.010
Maximal plaque (mm)	2.10 ± 0.79	2.22 ± 0.79	0.336
ABI,rt	1.19 ± 0.07	1.17 ± 0.08	0.688
ABI,lt	1.19 ± 0.08	1.16 ± 0.09	0.108

Data are expressed as mean ± SD

*GFR* glomerular filtration ratio, *LDL* low density lipoprotein, *HDL* high density lipoprotein, *BNP* Brain natriuretic peptide, *TG* triglyceride, *CK-MB* Creatine kinase-MB, *PWV* pulse wave velocity, *IMT* intima-medial thickness, *ABI* ankle-brachial index

^a^Analyzed by Mann–Whitney U test and data are expressed as median (IQR)

### Univariate and multivariate analyses (Table 5)

**Table 5:** Uni-and multivariate analysis of relating factors for positive result of ASE

	Odds ratio	95% CI	*p*
Univariate analysis			
Any abnormality on brain MRI	0.579	0.338–0.993	0.047
Age	0.952	0.927–0.978	< 0.001
BMI	0.924	0.855–0.998	0.045
Hypertension	0.566	0.336–0.953	0.032
Aspirin	0.117	0.015–0.892	0.038
E’	1.100	1.008–1.199	0.032
A’	1.170	1.023–1.338	0.022
S’	1.353	1.144–1.600	< 0.001
LV diastolic dysfunction	0.842	0.527–1.346	0.473
PWV	0.999	0.998–1.000	0.036
IMT	0.130	0.030–0.564	0.006
Carotid plaque	0.483	0.281–0.829	0.008
Multivariate analysis			
Any abnormality on brain MRI	0.878	0.451–1.709	0.701
age	0.975	0.936–1.017	0.241
BMI	0.903	0.821–0.994	0.036
Hypertension	0.817	0.428–1.557	0.539
Aspirin	0.179	0.022–1.470	0.109
E’	0.923	0.804–1.058	0.250
A’	1.280	1.048–1.563	0.015
S’	1.180	0.920–1.513	0.193
PWV	1.000	0.999–1.001	0.504
S’	1.159	0.901–1.491	0.250
IMT	0.216	0.039–1.190	0.078
Carotid plaque	0.634	0.327–1.231	0.634

*BMI* body mass index, *LV* left ventricular, *PWV* pulse wave velocity, *IMT* intima-medial thickness

Among the various parameters, univariate analysis was performed with parameters with a *p* value of less than 0.5. Univariate analysis showed that several clinical, echocardiographic, and other factors had predictive value for positive ASE. Younger age, lower BMI, lower prevalence of hypertension, less aspirin use, more favorable diastolic function, elevated Eʹ and Aʹ velocities, lower PWV, lower IMT, and lower presence of carotid plaque were associated with positive ASE. Multivariate analysis was performed with a parameter with a *p* value of less than 0.5 in univariate analysis.

Among variables found to be correlated with positive ASE, lower BMI (odds ratio [OR], 0.903, 95% confidence interval [CI], 0.821–0.994; *p* = 0.036) and higher Aʹ velocity (OR, 1.280; 95% CI, 1.048–1.563; *p* = 0.015) were independently correlated with positive ASE in patients with acute stroke or suspected stroke.

## Discussion

The novel findings of this study are that transthoracic ASE is an easy and convenient screening tool for PFO and that the greater the RSL degree, the higher the likelihood of PFO. Patients with positive ASE showed similar stroke severity and recurrence rate to those with negative ASE, but they were relatively younger, had a lower mean BMI, had a lower prevalence of hypertension, had less atherosclerosis, and had better cardiac performance. In addition, patients with positive ASE showed used much less aspirin.

### Characteristics of cryptogenic stroke and suggestive of PFO

Embolic stroke of undetermined source (ESUS) was introduced to identify patients with nonlacunar cryptogenic ischemic strokes in whom embolism was the likely stroke mechanism [17]. The characteristics of ESUS patients according to the review that emerged are of relatively young (compared with AF-associated stroke) patients with mild strokes and lower frequencies of conventional vascular risk factors than non-ESUS patients with ischemic stroke [18]. We also evaluated the clinical impact of PFO in patients < 65 years with cryptogenic stroke. The results were similar, suggesting that fewer conventional risk factors are found in patients with suspected PFO (as positive ASE), although the degree of severity was similar on brain MRI. TEE was performed in 21 consenting patients with negative ASE, all without PFO. On the other hand, PFO was confirmed in 36 of 51 (71%) positive ASE patients who underwent TEE (sensitivity of 100% and specificity of 58%). According to shunt grade, PFO was confirmed in all patients with RLS grade ≥ 2, but 15 (50%) of 30 patients with a grade 1 shunt demonstrated no PFO on TEE with ASE, which showed that the greater the RSL degree, the higher the likelihood of PFO. As suggested in previous study [16], the higher the RoPE score, the more likely the association between cryptogenic stroke and PFO. Although this study did not confirm PFO in all patients with positive ASE, the RoPE score was significantly higher in patients with positive ASE. The biggest drawback of this study is that the diagnosis rate of PFO according the results of ASE was not compared head to head by performing TEE in all patients.

### Clinical obstacles in implementing TEE

Since many patients in real clinical setting do not want to perform TEE, and TEE may not be needed in all patients in various economic and medical aspects, the results of ASE may be helpful in selectively implementing TEE in patients in need. Therefore, inferring the results of this study suggests that the higher the shunt grade of the transthoracic ASE, the higher the probability of stroke due to PFO, which may be considered for treatment. Reinthaler M. et al. recently published that PFO closure plus antiplatelets was superior in terms of stroke prevention compared to medical treatment alone, especially in patients with moderate to severe shunt and those younger than 45 years of age [18]. We then examined the cause of grade I shunt in transthoracic ASE without PFO on TEE with ASE. First, it is possible that the Valsalva maneuver was not exactly accurate at the time of TEE. It is extremely important to continue echocardiographic imaging during the strain and release phases of the Valsalva maneuver to prevent missing the diagnosis of a paradoxical shunt [19]. Second, the imaging resolution of TTE was insufficient; therefore, it is possible that an artifact in the LA was misunderstood as a few bubbles. Finally, a physiologic shunt (venous admixture) may have occurred in which a fraction of mixed venous blood does not become oxygenated as it moves from the RA to the LA and LV [20]. There were no significant differences in age, sex, or other risk factors between the patients with a grade 1 shunt with versus without PFO. In this study, we checked patients’ follow up to 2 years, and investigated recurrence of stroke in the meantime. Although not statistically significant, the recurrence rate was low in patients with positive ASE. Since the patients enrolled in this study were relatively younger patients with cryptogenic stroke (less than 65 years old), it is thought that the recurrence rate was low and the prognosis was not bad in both groups. And although there is no clear evidence for doing follow up better in patients with positive ASE patients than in patients with negative ASE patients, it might be due to patients’ more concerns about additional heart problem.

### Role of echocardiography in patients with stroke

The role of echocardiography in patients with stroke is not only to find structural heart disease or intracardiac mass but also to screen for PFO, especially in young patients who present with cryptogenic stroke and no cardiovascular risk factors [21]. Here we excluded patients with significant structural heart diseases or other abnormalities, so there were no significant intergroup differences in echocardiographic results. Nonetheless, diastolic performance was better in patients with positive ASE. In particular, Aʹ velocity of the mitral annulus using tissue Doppler imaging was slightly higher with statistical significance. Aʹ velocity is a good parameter for LA contractile function, and both systolic and diastolic function affect LA contractile function. A higher LV EF is associated with higher Aʹ velocity, while restrictive LV diastolic filling is associated with lower Aʹ velocity [22]. Although there was no significant intergroup difference in EF, this study also suggests that Aʹ velocity was significantly higher in patients with positive ASE because of higher Sʹ velocity and better diastolic performance.

### Other factors associated with stroke

Carotid atherosclerosis (carotid stiffness, IMT, and early plaque formation) is a marker of systemic atherosclerosis and a predictor of ischemic cerebrovascular disease (CVD) [23]. Increased carotid IMT and the presence of carotid plaques are well established predictors of CVD and ischemic stroke [24]. Likewise patients with a positive ASE had lower carotid IMT and fewer carotid plaques in this study.

It is well known that increased arterial stiffness (IAS) is an early marker of systemic atherosclerosis and has independent predictive value for cardiovascular events [25]. The PWV was also higher in patients with negative ASE.

### Role of transthoracic agitated saline echocardiography in treatment

A recent meta-analysis of randomized controlled trials of PFO showed that in selected patients with cryptogenic stroke, PFO closure is superior to medical therapy for preventing further strokes, which was particularly true for patients with moderate to large shunts [26]. The long-term follow-up data clarified that, with good patient selection, transcatheter PFO closure significantly reduces the risk of recurrent stroke compared with medical therapy in patients with cryptogenic stroke with no increased risk of serious adverse events or influence on major bleeding [27]. Therefore, as well as the presence of PFO, shunt grade is also an important consideration in the management of PFO with stroke, so it may be necessary to screen for PFO and confirm shunt grade using transthoracic ASE, which is more comfortable for the patient.

**Fig. 2:**
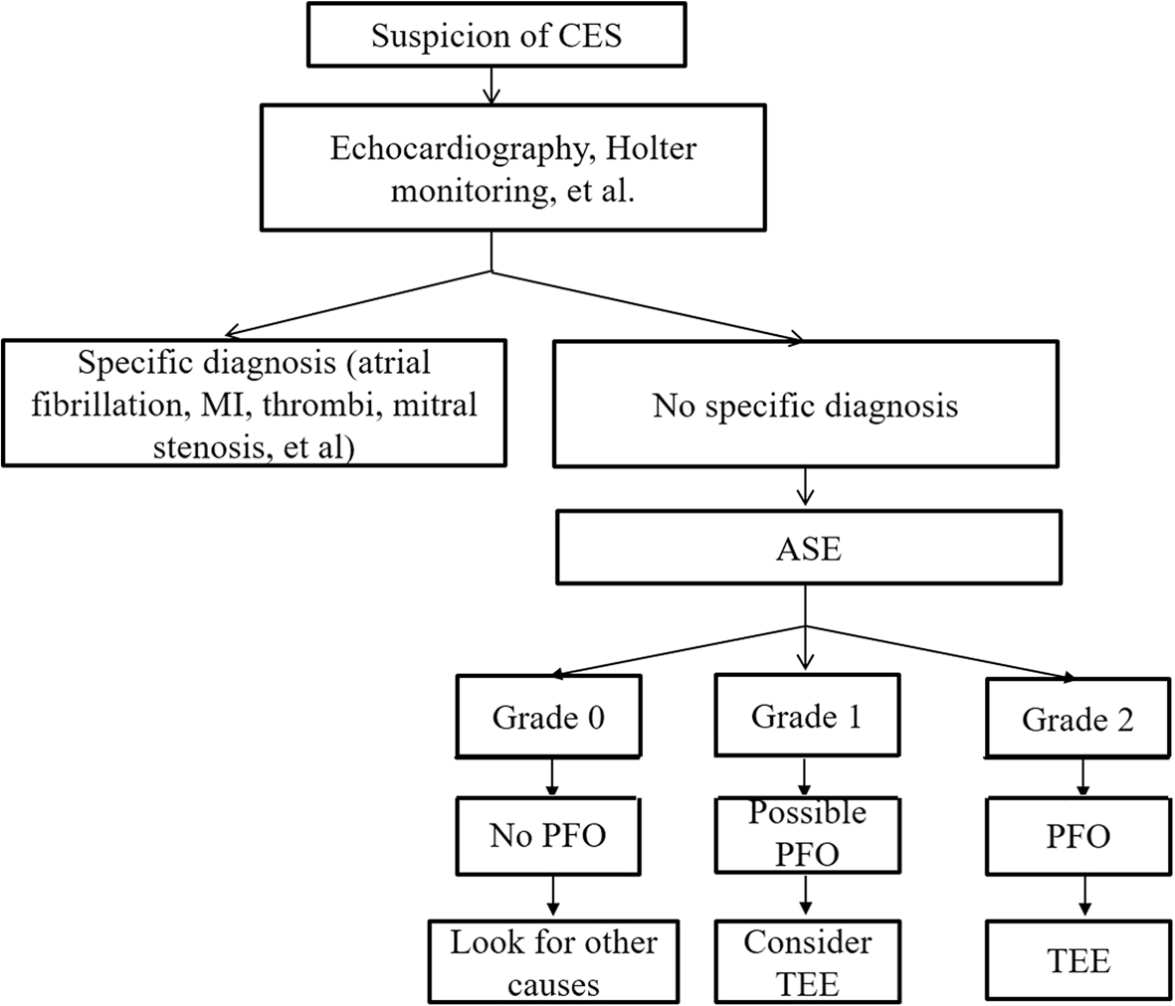
Suggested diagnostic algorithm for patients with suspected cardiac embolic stroke. CES: cardiac embolic stroke, MI: myocardial infarction, ASE: agitated saline echocardiography, PFO: patent foramen ovale, TEE: transesophageal echocardiography

Here, we suggest a diagnostic algorithm for patients with suspected cardiac embolic stroke according to our results (Fig. 2).

### Study limitations

TEE was not performed in many patients in this study. This was a major limitation of this study because of high possibility of selection bias; however, it was the main purpose of this study because most patients refuse TEE, transthoracic ASE was performed to screen for PFO and elucidate the clinical significance of the results. In addition, if TEE is impossible to do, we can consider other test, such as transcranial Doppler to confirm PFO in subsequent studies or clinical settings.

## Conclusions

In summary, transthoracic ASE is an easy and convenient screening tool for PFO, and the greater the degree of RSL, the higher the likelihood of PFO. Patients with positive ASE showed similar stroke severity to those with negative ASE, but they were relatively younger, had a lower mean BMI, had a lower prevalence of hypertension, less commonly had atherosclerosis, and had better cardiac performance. Our findings suggest that transthoracic ASE may be a good screening method for PFO and helpful in the diagnosis and treatment of PFO in patients with relatively low risk factors, good cardiac performance, and confirmed or suspected stroke.

## Data Availability

The whole images or part of it, neither has been published and is not being considered for publication elsewhere in whole or part in any language.

## Abbreviations

PFO: Patent foramen ovale
TEE: Transesophageal echocardiography
TCD: Transcranial Doppler
RLS: Right-to-left shunts
TTE: Transthoracic echocardiography
ASE: Agitated saline echocardiography
TIA: Transient ischemic attack
ABI: Ankle-brachial index
PWV: Pulse wave velocity
LV: Left ventricular
AF: Atrial fibrillation
PAD: Peripheral arterial disease
LV: Left ventricular
EDD: End-diastolic dimensions
IVSTd: End-diastolic interventricular septal thickness
EF: Ejection graction
LA: Left atrial
LVMI: LV mass index
TDI: Tissue Doppler image
GS: Global strain
IMT: Intima-media thickness
CCA: Common carotid artery
CB: Carotid bulb
ESUS: Embolic stroke of undetermined source
CVD: Cerebrovascular disease
IAS: Increased arterial stiffness
